# Depressive symptoms predict the incidence of common chronic diseases in women and men in a representative community sample

**DOI:** 10.1017/S0033291722000861

**Published:** 2023-07

**Authors:** Daniëlle Otten, Mareike Ernst, Antonia M. Werner, Ana N. Tibubos, Iris Reiner, Elmar Brähler, Jörg Wiltink, Matthias Michal, Markus Nagler, Philipp S. Wild, Thomas Münzel, Jochem König, Karl J. Lackner, Norbert Peiffer, Manfred E. Beutel

**Affiliations:** 1 Department of Psychosomatic Medicine and Psychotherapy, University Medical Center of the Johannes Gutenberg-University Mainz, Mainz, Germany; 2 Preventive Cardiology and Preventive Medicine – Department of Cardiology, University Medical Center of the Johannes Gutenberg-University Mainz, Mainz, Germany; 3 Center for Thrombosis and Hemostasis (CTH), University Medical Center of the Johannes Gutenberg-University Mainz, Mainz, Germany; 4 Department of Cardiology – Cardiology I, University Medical Center of the Johannes Gutenberg-University Mainz, Mainz, Germany; 5 Institute of Medical Biostatistics, Epidemiology and Informatics (IMBEI), University Medical Center of the Johannes Gutenberg-University Mainz, Mainz, Germany; 6 Institute of Clinical Chemistry and Laboratory Medicine, University Medical Center of the Johannes Gutenberg-University Mainz, Mainz, Germany; 7 German Center for Cardiovascular Research (DZHK), Partner Site Rhine-Main, Mainz, Germany; 8 Department of Ophthalmology, University Medical Center of the Johannes Gutenberg-University Mainz, Mainz, Germany

**Keywords:** Chronic disease, community sample, depressive symptoms, longitudinal study, somatic illnesses

## Abstract

**Background:**

Depression, the most frequent and harmful mental disorder, has been associated with specific somatic diseases as the leading cause of death. The purposes of this prospective study were to predict incident chronic diseases based on baseline depressive symptoms and to test sex-dependent effects.

**Methods:**

In a representative German community sample of over 12 000 participants, baseline depressive symptoms (assessed using the Patient Health Questionnaire-9) were tested as a predictor of new onset of cardiovascular disease (CVD), chronic obstructive lung disease, diabetes, cancer, and migraine at 5-year follow-up. To study disease incidence, we created subsamples for each chronic disease by excluding participants who already had the respective disease at baseline. Potential confounders were included in logistic regression models and sex-specific analyses were performed.

**Results:**

Controlling for demographic characteristics and loneliness, in men and women, baseline depressive symptoms were predictive of CVD, chronic obstructive lung disease, diabetes, and migraine, but not of cancer. When we additionally adjusted for metabolic and lifestyle risk factors, there was an 8% increase of chronic obstructive lung disease and migraine per point of depressive symptoms. There was a trend for CVD (4%; *p* = 0.053). Sex-sensitive analyses revealed trends for the relevance of depressive symptoms for CVD in men (*p* = 0.065), and for diabetes in women (*p* = 0.077).

**Conclusions:**

These findings underscore the need to implement screening for depression in the treatment of major somatic illnesses. At the same time, depressed patients should be screened for metabolic and lifestyle risk factors and for somatic diseases and offered lifestyle interventions.

## Introduction

Depression is one of the most frequent and harmful mental disorders with an estimated lifetime risk of 15–25%, affecting women about twice as frequently as men (Malhi & Mann, 2018). As it often takes a chronic course and is associated with elevated morbidity and mortality (Malhi & Mann, 2018), depression is an enormous public health concern. In aging societies, chronic diseases including cardiovascular disease (CVD), cancer, chronic respiratory disease, and diabetes have become the leading causes of death (Brennan, Perola, van Ommen, Riboli, & European Cohort Consortium, 2017). A growing body of research has identified close associations of depressive symptoms with chronic physical diseases. However, previous research has mostly focused on particular pairs of mental and medical diseases (Dijkstra-Kersten et al., 2017; Poole & Steptoe, 2018; Tibubos et al., 2019). In a national Danish registry study, Momen et al. (2020) reported a median hazard ratio of 1.37 for an association between 90 pairs of mental disorders and medical conditions. In the prospective English Longitudinal Study of Aging (ELSA), Poole and Steptoe (2018) found that depressive symptoms at baseline predicted a 5% increase (per point of depression, assessed with the CES-D) of incident chronic disease burden 10 years later.

CVDs include coronary artery disease (CAD), myocardial infarction (MI), peripheral arterial disease (PAD), stroke, and congestive heart failure (CHF). Previous studies showed (1) increased risk of CVD in depressed individuals, (2) heightened risk of depression following acute CVD, and (3) worse prognosis when CVD was complicated by depression (Khandaker et al., 2020; Penninx, 2017; Shao et al., 2020). The associations of depression with CVD were more pronounced in women than in men (Möller-Leimkühler, 2007).

With regard to chronic obstructive lung disease, several studies have shown higher prevalence rates of depression in chronic obstructive pulmonary disease (COPD) patients compared to the general population (Mikkelsen, Middelboe, Pisinger, & Stage, 2004; Putman-Casdorph & McCrone, 2009; van Manen et al., 2002). In this group, the prevalence of depression [Patient Health Questionnaire-9 (PHQ-9) ≥ 10] was twice as high (16.2% *v.* 7.5%) compared to participants without COPD (Ghaemi Kerahrodi et al., 2019). Also for patients with chronic bronchitis, a subgroup of COPD patients, the prevalence of depression was twice as high (15.9% *v.* 7.6%) compared to participants without chronic bronchitis (De Miguel Díez et al., 2011). Evidence supports a bidirectional association of asthma and depression (Choi et al., 2019). However, the effect of depression on asthma seems to be stronger than the other way around. An Egyptian literature review found that depression predicted the new onset of asthma in adults based on six studies which included 83 684 participants that were followed for 8–20 years, with 2334 cases of incident asthma in total (Fageeh et al., 2018). Only two studies reported an effect of asthma on incident depression. Furthermore, a review indicated an adverse effect of depression on the course of COPD (Laurin, Moullec, Bacon, & Lavoie, 2012), and another study described an adverse effect of depression on the course of asthma in older people (Patel, Patel, & Baptist, 2017). For COPD, this effect was not influenced by participants' sex (Laurin et al., 2012), but sex differences were not examined for asthma (Patel et al., 2017).

Diabetes mellitus and depression were often comorbid (Holt, de Groot, & Golden, 2014). For both type 1 and type 2 diabetes, the prevalence of depression was higher compared to the general population: it was three times as high in people with type 1 diabetes and twice as high in people with type 2 diabetes (Roy & Lloyd, 2012). The relation between type 2 diabetes and depression is mostly considered to be bi-directional (Egede & Ellis, 2010; Mezuk, Eaton, Albrecht, & Golden, 2008; Pan et al., 2010; Renn, Feliciano, & Segal, 2011). In one review, depression was associated with a 60% increased risk of type 2 diabetes, whereas type 1 diabetes was only associated with a 15% increased risk of depression (Mezuk et al., 2008). The prevalence of depression was higher in women who had diabetes than in men (Roy & Lloyd, 2012), which reflects the epidemiology of depression in the general population. Findings of sex-specific effects of depressive symptoms on diabetes mellitus are conflicting, indicating either no effects of sex (Mezuk et al., 2008) or only an effect for women (Demmer et al., 2015).

With regard to cancer, depression has been reported as a frequent consequence of the experience of this potentially life-threatening and increasingly chronic disease (Chochinov, 2001; Spiegel & Giese-Davis, 2003). It is compounded by pain and fatigue symptoms (Spiegel & Giese-Davis, 2003). Previous research has underscored the need to investigate associations of cancer and mental distress in a sex-sensitive way (Ernst et al., 2019). Evidence regarding the predictive value of depression for cancer is mixed. While associations of depression with cancer mortality (Lloyd-Williams, Shiels, Taylor, & Dennis, 2009; Pinquart & Duberstein, 2010; Satin, Linden, & Phillips, 2009; Spiegel & Giese-Davis, 2003) and cancer progression (Satin et al., 2009; Spiegel & Giese-Davis, 2003) were found, the contribution of depression to the etiology of cancer has remained a matter of debate (Gross, Gallo, & Eaton, 2010; Jia et al., 2017). Sex-specific investigations of the effects of depression on cancer incidence are scarce. Examining specific types of cancer, it was shown that for women, depression predicted new onset of breast cancer and for men, depression predicted new onset of prostate cancer (Gross et al., 2010).

Migraine, an episodic primary headache disorder (Vos et al., 2016), is considerably more prevalent in women than in men (Stovner et al., 2007) and has also been associated with depression (Breslau, Lipton, Stewart, Schultz, & Welch, 2003; Buse, Greisman, Baigi, & Lipton, 2019; Vetvik & MacGregor, 2017). Depression was predictive of new onset of (chronic) migraine (Ashina et al., 2012; Buse et al., 2019) and it was also a risk factor for increased migraine attacks (Victor et al., 2009) and chronic daily headaches (Buse et al., 2019; Wang, Fuh, Lu, & Juang, 2007). However, it is unclear whether depression is a predictive factor for migraine in both women and men. While triggering factors and attack thresholds for migraine are modulated by sex hormones (Vetvik & MacGregor, 2017), it is likely that the effects of depression on migraine differ for sex.

Furthermore, the new onset of diseases is shaped by sociodemographic, psychological, metabolic, and behavioral factors. For example, the incidence of chronic diseases increased with age (Ng, Sutradhar, Yao, Wodchis, & Rosella, 2020). Higher educational attainment reduced the risk of chronic lung diseases (Assari, Chalian, & Bazargan, 2020) and was negatively associated with the incidence of major cardiovascular events (Rosengren et al., 2019). In contrast, lower income and lower socioeconomic status (SES) increased the risk of chronic lung diseases (Assari et al., 2020) and were associated with a higher prevalence of migraine (Winter, Berger, Buring, & Kurth, 2011) and the risk of diabetes, also through health behaviors (Williams et al., 2010). Social isolation is a known risk factor for aggravating diseases or death rates (Holt-Lunstad, Smith, Baker, Harris, & Stephenson, 2015). Both social isolation and loneliness were found to be predictive of CVD and type 2 diabetes, even after controlling for negative affect, but they were not predictive of COPD and cancer (Christiansen et al., 2021). Loneliness was comparatively common among people with chronic headaches (Westergaard, Lau, Allesøe, Andreasen, & Jensen, 2021). Obesity increased the risk of CVD, diabetes [especially in combination with high body mass index (BMI)], cancer, and migraine (Ornello et al., 2015; Pereira, 2012; Wolin, Carson, & Colditz, 2010) and had negative effects on respiratory function (Peters, Suratt, Bates, & Dixon, 2018). Dyslipidemia also had negative effects on respiratory function (Peters et al., 2018). Additionally, dyslipidemia increased the risk of CVD and diabetes (Pereira, 2012; Petrie, Guzik, & Touyz, 2018) and has been linked to specific types of cancer (Fraeman, Nordstrom, Luo, Landis, & Shantakumar, 2013; Pothiwala, Jain, & Yaturu, 2009). Also hypertension was associated with cancer (Fraeman et al., 2013). Hypertension was furthermore a risk factor for diabetes and exacerbated CVD (Petrie et al., 2018), and was, according to some studies, associated with migraine (Wang & Wang, 2021). Elevated (especially high fastening) blood glucose is a known risk factor for cardiovascular events (Einarson, Machado, & Hemels, 2011). Lastly, smoking and physical inactivity have been linked to CVD, chronic respiratory disease, diabetes, cancer, and migraine (Hagen et al., 2018; Ng et al., 2020). Thus, empirical investigations that intend to contribute to a better understanding of the relationship between depression and major illnesses also need to consider these relevant – and potentially confounding – factors.

In fact, the association of depression and chronic diseases has been examined in a variety of cross-sectional and prospective studies using both population-based and patient samples, indicating that prevention and/or treatment of depressive symptoms might have beneficial effects for physical health, too. The aim of the present paper was to expand on this important public health issue. As the relation of depressive symptoms and chronic illness has been most extensively studied in CVDs and to a lesser extent in other chronic diseases, we investigated five common conditions within the same population sample. While the morbidity of chronic somatic and mental diseases differs considerably between men and women, sex differences in the association of mental and somatic diseases have hardly been systematically explored. Therefore, we used sex-specific analyses. We also controlled for relevant sociodemographic, somatic, and lifestyle risk factors of the respective diseases that could constitute confounding variables.

In summary, the purposes of this prospective study are:

1. to predict *incident chronic disease* based on depressive symptoms regarding five major diseases (CVD, chronic obstructive lung disease, diabetes mellitus, cancer, and migraine) in the German population from baseline to 5-year follow-up and
2. to examine whether the effects of depressive symptoms at baseline on the onset of chronic disease 5 years later differ between women and men.

## Methods

### Procedure and study sample

The Gutenberg Health Study is a population-based, prospective, observational single-center cohort study in the Rhine-Main-Region, Germany (Beutel et al., 2020; Hohn et al., 2015; Wild et al., 2012). Its aim is to improve the individual risk prediction for diseases. The project focuses on several diseases, such as CVDs, metabolic diseases, diseases of the immune system, eye diseases, and mental disorders. The study protocol and documents were approved by the local ethics committee of the Medical Chamber of Rhineland-Palatinate and the local data safety commissioner. All study investigations have been conducted in line with the Declaration of Helsinki and principles outlined in recommendations for Good Clinical Practice and Good Epidemiological Practice. Research was performed in accordance with all regulations. Participants were included after giving informed consent. Exclusion took place in case of inability to participate due to psychological and physical impairments or insufficient knowledge of the German language. The sample was drawn randomly from the local population registry in the city of Mainz and the district of Mainz-Bingen, stratified 1:1 for sex and residence and in equal strata for decades of age (the included age range was 35–74 years). The response, defined as the recruitment efficacy proportion, was 60.3%. At baseline (2007–2012), a total of *N* = 15 010 participants were included. Of these participants, *N* = 12 422 (82.8%) participated in the follow-up study (2012–2017).

For this study, only participants who participated in the baseline and follow-up assessment were included. Furthermore, participants with missing values in the baseline depression assessment were excluded. This led to a sample of *N* = 12 285 with a mean age of 54.4 (±10.9) at baseline; including 6005 women (48.9%) and 6280 men (51.1%). In order to study disease incidence, we created subsamples for each chronic disease by excluding participants who already had the respective disease at baseline. They were, however, included in the analyses statistically predicting the onset of the other diseases. The subsamples consisted of more than 10 000 participants each: CVD: *N* = 11 049; chronic obstructive lung disease: *N* = 11 681; diabetes: *N* = 11 333; cancer: *N* = 11 228; and migraine: *N* = 11 452.

### Materials and assessment

The 5-h baseline examination in the study center comprised of an evaluation of classical cardiovascular risk factors and clinical variables, a computer-assisted personal interview, laboratory examinations from a venous blood sample, blood pressure and anthropometric measurements. All examinations were performed according to standard operating procedures by certified medical technical assistants.

### Measures

#### Chronic diseases

CVD included CAD, MI, PAD, stroke, and CHF. The presence of these diseases was assessed by inquiring whether participants had previously been diagnosed with the respective disease by a physician. A confirmative answer for at least one of the diseases indicated the presence of CVD. The question was similar in the baseline and follow-up assessment: at baseline, it referred to the complete medical history, whereas at follow-up, it referred to the period since the baseline examination. Cancer was assessed in the same way. Chronic obstructive lung disease was assessed by inquiring about medicated asthma or medicated chronic bronchitis. Diabetes mellitus was defined as self-reported history of diabetes, corresponding medical therapy, or fasting blood glucose ≥126 mg/dL or non-fasting blood glucose ≥200 mg/dL. Migraine was assessed with the question ‘Did you have migraine in the last 12 months?’. Migraine was defined as regular attacks over a period of at least 1 year.

#### Depressive symptoms

Depressive symptoms were measured with the depression module of the PHQ-9. It assesses the frequency of the nine diagnostic criteria of major depression according to DSM-V (American Psychiatric Association, 2013). Using a Likert scale ranging from 0 = not at all to 3 = nearly every day, participants are asked to indicate how often they were bothered by the respective symptom over the course of the last 2 weeks. The sum score ranges from 0 to 27. Clinically relevant symptom burden was defined as a sum score ≥10. Löwe et al. (2004) found a sensitivity of 81% and a specificity of 82% for depressive disorders determined by this cut-off. Additionally, the PHQ-9 was confirmed as a reliable and unidimensional measure for depression (Kocalevent, Hinz, & Brähler, 2013). Within the present sample, its internal consistency was good (Cronbach's *α* = 0.80).

#### Sociodemographic factors

Sociodemographic variables were assessed via self-report. These included: participants' sex, age in years, SES, living with partner (no/yes), and living alone. SES was defined as an index ranging from 3 (lowest SES) to 21 (highest SES) based on education, profession, and income following Lampert, Kroll, Müters, and Stolzenberg (2013).

#### Psychological factors

Loneliness was assessed by a single item ‘I am frequently alone/have few contacts’ rated as 0 = no, does not apply; 1 = yes it applies, but I do not suffer from it; 2 = yes, it applies, and I suffer slightly; 3 = yes, it applies, and I suffer moderately; 4 = yes, it applies, and I suffer strongly (Beutel et al., 2017).

#### Metabolic factors

This study included the metabolic factors: BMI, dyslipidemia, obesity, blood glucose, and hypertension. BMI was calculated by dividing weight (kg) through height (m^2^). Obesity was defined as a BMI ≥30 kg/m^2^. A diagnosis of dyslipidemia was based on the current intake of lipid-modifying drugs or an low-density lipoprotein (LDL)/high-density lipoprotein (HDL) ratio >3.5. Blood glucose was measured by HbA1C. Hypertension was assessed by mean systolic blood pressure (≥140 mm Hg) or mean diastolic blood pressure (≥90 mm Hg) or use of antihypertensive medications.

#### Lifestyle factors

As lifestyle factors, smoking and physical activity were included. Smoking was dichotomized into non-smokers (combining never smokers and ex-smokers) and smokers (≥1 cigarette/day). Physical activity was inquired with the Short QUestionnaire to ASsess Health-enhancing physical activity (SQUASH; InterAct Consortium et al., 2012). Participants were asked to report about their regular physical activity during an average week over the past year. The SQUASH captures four common domains of physical activity: commuting, household (domestic) work, leisure-time, and work activities. Physical activity was measured based on its frequency (days per week), duration (average time per day), and effort (light/moderate/intense). Sleeping, lying, sitting, and standing were classified as inactivity. Each domain of physical activity was assigned a MET value (Ainsworth et al., 2000). Furthermore, an intensity score (ranging from 1 to 9) (Wendel-Vos, Schuit, Saris, & Kromhout, 2003) and the total minutes of activity per week were calculated. This information was used to create an activity score, reported as total minutes of activity per week × intensity score. For interpretability reasons, this value was divided by 1000, i.e. regression coefficients for physical activity represent a 1000-units increase in activity score.

### Statistical analyses

Descriptive statistics were performed as absolute and relative proportions for categorical data, means, and standard deviations for continuous variables and median with interquartile range (if not fulfilling normal distribution). Inference tests between depression and no depression were calculated with *t* tests or χ^2^ tests. We performed multivariate logistic regressions in order to ascertain whether depressive symptoms predicted the incidence of chronic diseases. The investigated chronic diseases were modeled as dichotomous dependent variables. For each disease, two models were tested: in model a, we entered depression (depressive symptoms based on the PHQ-9 sum score), sex, interaction of depression with sex, sociodemographic characteristics (age, SES, living with partner, living alone), and the psychological characteristic loneliness. Model b additionally included metabolic factors (BMI, dyslipidemia, obesity, blood glucose, hypertension) and lifestyle factors (physical activity, smoking).

## Results

### Participant characteristics

Descriptive statistics, stratified for the presence of depression at baseline, are displayed in Table 1. Of the total sample of 12 285 participants, 873 were depressed (i.e. they surpassed the PHQ-9 ≥ 10 cut-off). With regard to sociodemographic factors, depressed participants were more likely to be women, younger, not living with a partner, living alone, had a lower SES. More of them also indicated feeling lonely. Concerning metabolic factors, depressed individuals had a higher BMI and more of them had dyslipidemia and obesity. No differences were found with respect to blood glucose levels and hypertension. Additionally, depressed participants were more likely to be smokers than non-depressed participants, but there were no differences concerning physical activity.

**Table 1.** Characteristics of participants at baseline

	Total sample	No depression (PHQ-9 ⩽ 10)	Depression (PHQ-9 ⩾ 10)	*p*-Value
Sociodemographic				
Sex (women)	6005 (48.9%)	5487 (48.1%)	518 (59.3%)	<0.001
Age	54.4 ± 10.9	54.6 ± 10.9	52.4 ± 9.9	<0.001
Living with partner	10 114 (82.3%)	9511 (83.4%)	603 (69.1%)	<0.001
Living alone	1637 (13.5%)	1435 (12.7%)	202 (24.0%)	<0.001
SES	13.3 ± 4.4	13.4 ± 4.4	12.4 ± 4.2	<0.001
Psychological				
Loneliness	1188 (9.7%)	866 (7.6%)	322 (37.4%)	<0.001
Metabolic				
BMI	26.4 (23.8–29.7)	26.3 (23.8–29.6)	27.4 (24.3–30.9)	<0.001
Dyslipidemia	4060 (33.1%)	3722 (32.7%)	338 (38.7%)	<0.001
Obesity	2875 (23.4%)	2607 (22.8%)	268 (30.8%)	<0.001
Blood glucose	5.5 (5.2–5.8)	5.50 (5.2–5.8)	5.50 (5.2–5.8)	0.150
Hypertension	5869 (47.8%)	5468 (47.9%)	401 (46.1%)	0.310
Behavioral				
Smoking	2199 (17.9%)	1978 (17.4%)	221 (25.3%)	<0.001
Physical activity	7.2 (5.2–9.4)	7.2 (5.1–9.4)	7.4 (5.5–9.7)	0.063

*Note*. Descriptive statistics were performed as absolute and relative proportions for categorical data, means, and standard deviations for continuous variables and median with interquartile range if not fulfilling normal distribution.

### New onset of diseases

The numbers of cases for new onset of disease was *N* = 444 for CVD, *N* = 308 for chronic obstructive lung disease, *N* = 447 for diabetes mellitus, *N* = 569 for cancer, and *N* = 353 for migraine. The incidence proportions at 5-year follow-up were 4.0% for CVD, 2.6% for chronic obstructive lung disease, 3.9% for diabetes mellitus, 5.1% for cancer, and 3.1% for migraine.

### Main analyses

We performed several multiple logistic regression analyses in order to examine the effect of depressive symptoms on new onset of CVD, chronic obstructive lung disease, diabetes mellitus, cancer, and migraine. Table 2 reports coefficients of the predictor *depressive symptoms at baseline*. Based on the respective model a (adjusted for socioeconomic factors and loneliness), baseline depressive symptoms predicted incidence of CVD, chronic obstructive lung disease, diabetes mellitus, and migraine at follow-up in the total sample and in the subsamples of women and men. In the respective model b (additionally adjusted for metabolic and lifestyle factors), depressive symptoms were still predictive of chronic obstructive lung disease and migraine in the total sample and in women and men. A one-unit increase in depressive symptoms at baseline increased the risk for the onset of the respective disease by 5–8%.^†^^1^ Associations with CVD in men (*p* = 0.065) and with diabetes mellitus in women (*p* = 0.077) only reached trend level, not statistical significance.

**Table 2.** Results of multiple logistic regression models of new onset of CVD, chronic obstructive lung disease, diabetes mellitus, cancer, and migraine on depressive symptoms at baseline

	CVD				Chronic obstructive lung disease				Diabetes mellitus				Cancer				Migraine			
	OR	CI	*p*	Nag. *R*^2^	OR	CI	*p*	Nag. *R*^2^	OR	CI	*p*	Nag. *R*^2^	OR	CI	*p*	Nag. *R*^2^	OR	CI	*p*	Nag. *R*^2^
Total																				
Model a	**1.06**	1.02–1.10	0.003	0.110	**1.08**	1.04–1.13	<0.001	0.062	**1.05**	1.02–1.09	0.005	0.074	1.02	0.98–1.05	0.390	0.082	**1.07**	1.03–1.13	0.003	0.100
Model b	1.04	1.00–1.09	0.053	0.360	**1.08**	1.03-1.13	0.001	0.310	1.03	0.98–1.07	0.220	0.500	1.02	0.98–1.06	0.270	0.310	**1.08**	1.03–1.14	0.001	0.280
Women																				
Model a	**1.05**	1.01–1.10	0.019	0.120	**1.08**	1.03–1.12	<0.001	0.073	**1.06**	1.02–1.10	0.007	0.088	1.02	0.98–1.06	0.260	0.050	**1.05**	1.02–1.09	0.004	0.096
Model b	1.03	0.98–1.09	0.240	0.430	**1.06**	1.01–1.12	0.017	0.380	1.05	0.99–1.10	0.077	0.540	1.01	0.97–1.06	0.610	0.320	**1.05**	1.01–1.09	0.019	0.290
Men																				
Model a	**1.06**	1.02–1.10	0.002	0.099	**1.08**	1.03–1.13	0.002	0.052	**1.05**	1.01–1.09	0.006	0.059	1.02	0.98–1.06	0.260	0.050	**1.06**	1.01–1.12	0.017	0.061
Model b	1.04	1.00–1.09	0.065	0.320	**1.07**	1.02–1.12	0.008	0.220	1.03	0.99–1.08	0.170	0.470	1.03	0.99–1.08	0.110	0.310	**1.07**	1.02–1.13	0.010	0.230

OR, odds ratio; CI, confidence interval (2.5–97.5%); Nag. *R*^2^, Nagelkerke *R*^2^.

*Note*. For statistically significant predictors, the OR is printed in bold. Model a: adjusted for sex and interaction of depression with sex (only total sample), sociodemographic characteristics (age, social economic status, living with partner, living alone), and loneliness (psychological factor). Model b: adjusted for sex and interaction of depression with sex (only total sample), sociodemographic characteristics (age, social economic status, living with partner, living alone), loneliness (psychological factor), metabolic factors (BMI, dyslipidemia, obesity, blood glucose, hypertension), and lifestyle factors (physical activity, smoking).

Full models are displayed in the online Supplementary material. Online Supplementary Table S1 displays the full model for the complete sample; online Supplementary Tables S2*a* and *b* display the full model separately for women and men. Male sex was an important predictor of CVD and diabetes mellitus; and female sex of migraine. Interactions between depressive symptoms and sex were not statistically significant. Higher age was a predictor of CVD and cancer, whereas lower age was a predictor of migraine. For men, lower age additionally predicted diabetes mellitus. Lower SES was a predictor of diabetes and migraine in the whole sample. Sex-specific models revealed an effect of SES on chronic diseases (CVD and migraine) only in women. Higher BMI predicted CVD, chronic obstructive lung disease, and diabetes. However, for men, there was no effect of BMI on chronic obstructive lung disease. Dyslipidemia was a significant predictor of diabetes mellitus, but in sex-specific analyses, this only applied to men. Blood glucose levels and hypertension were significant predictors of diabetes mellitus and migraine. Sex-specific models, however, revealed an effect of blood glucose on migraine only for men and an effect of hypertension on migraine only for women. Smoking was a significant predictor for CVD and chronic obstructive lung disease, although in sex-specific analyses, the effect of smoking on chronic obstructive lung disease was not significant in men. Lastly, physical activity predicted diabetes mellitus only in men.

## Discussion

In this study, we examined the associations of depressive symptoms and new onset of the major chronic somatic diseases, CVD, chronic obstructive lung disease, diabetes mellitus, cancer, and migraine over a course of 5 years within a representative adult community cohort including participants aged 35–74 years. The statistical models included tests of sex-specific effects and the most relevant confounding variables (from different domains of life) of the associations of interest.

When adjusted for demographic data and loneliness, in men and women, baseline depression was predictive of CVD, chronic obstructive lung disease, diabetes mellitus, and migraine, but not of cancer. When we additionally adjusted for metabolic and lifestyle baseline risk factors, there was an 8% increase of chronic obstructive lung disease and migraine per point of depressive symptoms. The association with CVD showed only a trend, and no associations were found with diabetes mellitus and cancer. There were no statistically significant interactions of depression and sex in the prediction of disease. However, when women and men were analyzed separately, in addition to the predictive value of depressive symptoms on chronic obstructive lung disease and migraine, a trend for the prediction of CVD was found in men only, and a trend for the prediction of diabetes was found in women only.

The present findings extend the existing prospective research on the association between depression and chronic somatic disease from CVD to other important chronic diseases, which are also associated with diminished quality of life. The statistically significant effects on chronic obstructive lung disease, CVD, and the negative findings regarding diabetes and cancer are in line with previous reports from the ELSA study (Poole & Steptoe, 2018), a population-based British study of healthy adults aged 50 and over. The strength of the observed associations was similar, too. Additionally, our results are consistent with investigations of a national Danish registry study including almost 6 million patients which indicated increased circularly, endocrine, pulmonary conditions, but not cancer in up to 15 years following the diagnosis of depression (Momen et al., 2020). We extended previous research by carefully conducting sex-specific analyses, both in the form of interaction terms and sex-specific analyses.

Comorbid medical and mental conditions may be influenced by pre-existing adverse factors, such as shared environmental risk factors, socioeconomic disadvantage, substance use, childhood maltreatment, or shared genetic factors. At the baseline assessment, depression was associated with female sex, lower age, sociodemographic disadvantage, living without a partner, living alone, and a considerably higher rate of loneliness. Additionally, depressed individuals were more likely to be smokers. In the following, the observed associations *over time* corroborate previous research which found that effects of mental disorders on medical illness are likely to be mediated by SES and lifestyle factors (Momen et al., 2020). They were also relevant predictors for CVD, chronic obstructive lung disease, and diabetes in this study. However, there were no significant effects of loneliness, living without a partner, or living alone on the new onset of any of the chronic diseases. This finding contrasts a Danish study with a similar age range that reported longitudinal associations of loneliness and social isolation with CVD and diabetes (Christiansen et al., 2021). However, the differential results could be explained by commonalities shared by depression and loneliness, and the predictive value of loneliness for depression itself (Cacioppo, Hawkley, & Thisted, 2010; Wang, Mann, Lloyd-Evans, Ma, & Johnson, 2018). Furthermore, depressed individuals had more metabolic (BMI, obesity, dyslipidemia) risk factors at baseline, which is in line with previous studies. Associations between obesity and new onset of chronic diseases were not found, which contradicts previous findings (Pereira, 2012). While current research found dyslipidemia to be a risk factor for diabetes and CVD (Petrie et al., 2018), we only found an effect of diabetes in men. While their interaction is not fully understood, these risk factors are plausible links to new onset of somatic disease. Discussed pathways include e.g. via stress (Yaribeygi, Panahi, Sahraei, Johnston, & Sahebkar, 2017) and sympatho-adrenergic activation (social factors), metabolic or inflammatory pathways (Liu, Wang, & Jiang, 2017) which played a role in linking depression and CVD (Shao et al., 2020), or direct toxic effects (such as smoking). Our study also indicated differential associations between depressive symptoms and major illnesses; and it suggested that lifestyle factors were of varying relevance: in the models of CVD and diabetes, the effects of depressive symptoms were diminished once physical activity and smoking were included in the analyses (in addition to the metabolic factors). Interestingly, the effects of depressive symptoms on chronic obstructive lung disease remained statistically significant after including smoking. Smoking was previously highlighted as a strong risk factor for the onset of asthma in several reviews and meta-analyses (McLeish & Zvolensky, 2010) and chronic bronchitis (Axelsson et al., 2016; Forey, Thornton, & Lee, 2011). Thus, besides prevention and intervention efforts directly aimed at promoting mental health, support of smoking cessation could be an important measure to counteract the development of chronic physical illnesses in the community, especially in individuals with mental illness.

Screening for depressive symptoms has been established in medical treatment guidelines for chronic diseases to various degrees [e.g. for cardiovascular (Michal et al., 2013) or oncological disorders (Ernst et al., 2019; Saracino & Nelson, 2019)]. The present findings also suggest the need to implement screening for chronic obstructive lung disease, diabetes, and migraine. At the same time, depressed patients should be screened for modifiable risk factors such as metabolic and lifestyle factors, and for somatic diseases. Within the context of primary care or counseling/psychotherapy, depressed individuals who seek support could also be offered lifestyle interventions, especially supporting smoking cessation.

### Strengths and limitations

The strengths of our study are the large size of a population-based sample and the statistical control of important confounding variables, including demographic, metabolic, and lifestyle factors. This analytic approach must be considered conservative as it also included potential mediators between depressive symptoms and somatic disease, reducing the associations of interest.

While we expect self-reported diagnoses to be reliable (Poole & Steptoe, 2018), it is a limitation that incident somatic diagnoses were mainly based on self-report. Additionally, the assessment of migraine was imprecise. While chronic migraine should be measured according to the number of times a typical migraine headache occurs within a month, we only assessed whether someone suffered from migraine in the previous 12 months. Information about depressive symptoms was also gathered via self-report (instead of, e.g. operationalized as a formal diagnosis). Research has found strong, statistically significant correlations between clinician-rated instruments and self-report assessments for depression exist (Domken, Scott, & Kelly, 1994). Comparing the PHQ-9 with semi-structured diagnostic interviews, studies found that sensitivity for major depression was similar, especially for slightly older adult populations (Levis, Benedetti, & Thombs, 2019). The present study assessed depression at baseline, when participants' age ranged between 35 and 74 years. Mental disorders most frequently start in young adulthood (when individuals are in their 20s or 30s), and we do not know depressed participants' age at onset of depression in the present sample, or who experienced a chronic course of depression. As previous research showed that the cumulative incidences of diagnosis of medical conditions within 15 years were higher among persons diagnosed with a mental disorder at a younger age (Momen et al., 2020), the present operationalization may have underestimated the association of depression with subsequent somatic illness. Any interpretation of the findings also needs to consider that we investigated the new onset of somatic disease, and we excluded one-third of participants who already suffered from chronic disease at baseline. Furthermore, comorbidity exists between the diseases examined in this study, for example between chronic obstructive lung disease (e.g. COPD) and CVD (Mannino, Thorn, Swensen, & Holguin, 2008; Rabe, Hurst, & Suissa, 2018), and diabetes (Mannino et al., 2008). Besides, there are also individuals who do have one of the studied chronic diseases, but have not yet received a formal diagnosis. Furthermore, unlike Poole and Steptoe (2018) who used data of a 10-year follow-up and Momen et al. (2020) who investigated up to 15-year follow-up data, we were limited to a 5-year follow-up.

## Footnotes

† The notes appear after the main text.

## Note

1 The relatively low incidence proportions allow approximation of risk by the odds ratios.

## Supplementary material

For supplementary material accompanying this paper visit https://doi.org/10.1017/S0033291722000861. click here to view supplementary material
