# Supplementary material for: Depressive symptoms predict the incidence of common chronic diseases in women and men in a representative community sample
Source: Psychol Med. 2022 Apr 21;53(9):4172–80. doi: 10.1017/S0033291722000861 (PMC10317822; doi:10.1017/S0033291722000861)
Supplement: Supplementary file 1 [file S0033291722000861sup.zip › S0033291722000861sup001.docx]

**Depressive symptoms predict the incidence of common chronic diseases in women and men in a representative community sample**

Daniëlle Otten^1^, Mareike Ernst^1^, Antonia M. Werner^1^, Ana N. Tibubos^1^, Iris Reiner^1^, Elmar Brähler^1^, Jörg Wiltink^1^, Matthias Michal^1^, Markus Nagler^2^, Philipp S. Wild^2,3,7^, Thomas Münzel^4,7^, Jochem König^5^, Karl J. Lackner^6,7^, Norbert Peiffer^8^, Manfred E. Beutel^1^

^1^ Department of Psychosomatic Medicine and Psychotherapy, University Medical Center of the Johannes Gutenberg-University Mainz, Mainz, Germany

^2^ Preventive Cardiology and Preventive Medicine – Department of Cardiology, University Medical Center of the Johannes Gutenberg-University Mainz, Mainz, Germany

^3^ Center for Thrombosis and Hemostasis (CTH), University Medical Center of the Johannes Gutenberg-University Mainz, Mainz, Germany

^4^ Department of Cardiology – Cardiology I, University Medical Center of the Johannes Gutenberg-University Mainz, Mainz, Germany

^5^ Institute of Medical Biostatistics, Epidemiology and Informatics (IMBEI), University Medical Center of the Johannes Gutenberg-University Mainz

^6^ Institute of Clinical Chemistry and Laboratory Medicine, University Medical Center of the Johannes Gutenberg-University Mainz, Mainz, Germany

^7^ German Center for Cardiovascular Research (DZHK), partner site Rhine-Main, Mainz, Germany

^8^ Department of Ophthalmology, University Medical Center of the Johannes Gutenberg-University Mainz, Mainz, Germany

*Correspondence:*
M.Sc. Daniëlle Otten
Department of Psychosomatic Medicine and Psychotherapy
University Medical Center of the Johannes Gutenberg-University Mainz
Langenbeckstraße 1, 55131 Mainz, Germany
Phone: +49 (0)6131 17-7643
E-Mail: [Danielle.Otten@unimedizin-mainz.de](mailto:Danielle.Otten@unimedizin-mainz.de)

Supplementary Table 1. Results of multiple logistic regression models of new onset of CVD, chronic obstructive lung disease, diabetes mellitus, cancer, and migraine on depressive symptoms at baseline.

|  |  |  |  |  |  |  |  |  |  |  | |  |  |  | |  |  |
| --- | --- | --- | --- | --- | --- | --- | --- | --- | --- | --- | --- | --- | --- | --- | --- | --- | --- |
|  | CVD | | | Chronic obstructive  lung disease | | | Diabetes mellitus | | | Cancer | | | | Migraine | | | |
|  | OR | CI | *p* | OR | CI | *p* | OR | CI | *p* | OR | | CI | *p* | OR | | CI | *p* |
|  |  |  |  |  |  |  |  |  |  |  | |  |  |  | |  |  |
| Depressive symptoms | 1.04 | 1.00-1.09 | .053 | **1.08** | 1.03-1.13 | .001 | 1.03 | 0.98-1.07 | .220 | 1.02 | | 0.98-1.06 | .270 | **1.08** | | 1.03-1.14 | .001 |
| Sex (women) | **0.57** | 0.39-0.82 | .003 | 1.25 | 0.82-1.91 | .300 | **0.54** | 0.37-0.81 | .002 | 0.91 | | 0.67-1.24 | .550 | **2.79** | | 1.88-4.14 | <.001 |
| Depressive symptoms*sex | 0.99 | 0.93-1.06 | .770 | 0.98 | 0.91-1.04 | .440 | 1.03 | 0.97-1.10 | .370 | 1.00 | | 0.95-1.06 | .980 | 0.96 | | 0.90-1.02 | .200 |
| *Sociodemographic* |  |  |  |  |  |  |  |  |  |  | |  |  |  | |  |  |
| Age | **1.08** | 1.06-1.09 | <.001 | 1.01 | 1.00-1.03 | .100 | 0.99 | 0.97-1.00 | .072 | **1.05** | | 1.04-1.07 | <.001 | **0.95** | | 0.94-0.97 | <.001 |
| SES | 0.99 | 0.96-1.02 | .370 | 0.99 | 0.95-1.02 | .410 | **0.97** | 0.94-1.00 | .045 | 1.01 | | 0.99-1.04 | .220 | **0.96** | | 0.93-0.99 | .008 |
| Living with partner | 0.87 | 0.47-1.60 | .650 | 1.04 | 0.52-2.09 | .900 | 0.79 | 0.44-1.44 | .450 | 1.49 | | 0.76-2.94 | .250 | 1.05 | | 0.63-1.75 | .840 |
| Living alone | 0.75 | 0.38-1.45 | .390 | 1.18 | 0.56-2.48 | .660 | 0.79 | 0.41-1.50 | .470 | 1.57 | | 0.77-3.20 | .220 | 0.90 | | 0.50-1.61 | .720 |
| *Psychological* |  |  |  |  |  |  |  |  |  |  | |  |  |  | |  |  |
| Loneliness | 1.10 | 0.73-1.67 | .650 | 0.98 | 0.63-1.54 | .940 | 1.21 | 0.80-1.83 | .370 | 0.95 | | 0.65-1.38 | .790 | 1.12 | | 0.76-1.63 | .570 |
| *Metabolic* |  |  |  |  |  |  |  |  |  |  | |  |  |  | |  |  |
| BMI | **1.08** | 1.05-1.12 | <.001 | **1.05** | 1.01-1.09 | .021 | **1.07** | 1.04-1.11 | <.001 | 1.01 | | 0.98-1.05 | .450 | 1.04 | | 1.00-1.08 | .064 |
| Dyslipidemia | 1.25 | 0.99-1.57 | .063 | 1.26 | 0.95-1.69 | .110 | **1.43** | 1.12-1.83 | .004 | 1.12 | | 0.91-1.38 | .290 | 0.94 | | 0.70-1.28 | .700 |
| Obesity | 0.78 | 0.54-1.14 | .200 | 0.97 | 0.62-1.53 | .900 | 1.27 | 0.88-1.84 | .200 | 1.00 | | 0.71-1.41 | .990 | 0.66 | | 0.41-1.05 | .081 |
| Blood glucose | 1.06 | 0.90-1.26 | .480 | 0.89 | 0.70-1.13 | .330 | **51.41** | 34.13-77.43 | <.001 | 1.00 | | 0.85-1.17 | .960 | **0.73** | | 0.56-0.94 | .014 |
| Hypertension | 1.21 | 0.93-1.56 | .150 | 1.11 | 0.82-1.50 | .490 | **1.76** | 1.33-2.33 | <.001 | 1.13 | | 0.91-1.41 | .280 | **0.72** | | 0.53-0.96 | .027 |
| *Lifestyle* |  |  |  |  |  |  |  |  |  |  | |  |  |  | |  |  |
| Physical activity | 1.02 | 0.99-1.05 | .240 | 1.01 | 0.97-1.04 | .690 | 0.98 | 0.95-1.01 | .140 | 0.98 | | 0.96-1.01 | .240 | 0.98 | | 0.95-1.02 | .350 |
| Smoking | **2.04** | 1.55-2.70 | <.001 | **1.71** | 1.25-2.35 | <.001 | 1.17 | 0.87-1.58 | .300 | 0.88 | | 0.66-1.17 | .370 | 0.96 | | 0.71-1.29 | .790 |
|  |  |  |  |  |  |  |  |  |  |  | |  |  |  | |  |  |
|  | Nagelkerke *R^2^*=.360 | | | Nagelkerke *R^2^*=.310 | | | Nagelkerke *R^2^*=.500 | | | | Nagelkerke *R^2^*=.310 | | | | Nagelkerke *R^2^*=.280 | | |

Note: OR=odds ratio; CI=confidence interval (2,5%-97,5%). For statistically significant predictors, the OR is printed in bold.
