# Supplementary material for: Depressive symptoms predict the incidence of common chronic diseases in women and men in a representative community sample
Source: Psychol Med. 2022 Apr 21;53(9):4172–80. doi: 10.1017/S0033291722000861 (PMC10317822; doi:10.1017/S0033291722000861)
Supplement: Supplementary file 1 [file S0033291722000861sup.zip › S0033291722000861sup002.docx]

**Depressive symptoms predict the incidence of common chronic diseases in women and men in a representative community sample**

Daniëlle Otten^1^, Mareike Ernst^1^, Antonia M. Werner^1^, Ana N. Tibubos^1^, Iris Reiner^1^, Elmar Brähler^1^, Jörg Wiltink^1^, Matthias Michal^1^, Markus Nagler^2^, Philipp S. Wild^2,3,7^, Thomas Münzel^4,7^, Jochem König^5^, Karl J. Lackner^6,7^, Norbert Peiffer^8^, Manfred E. Beutel^1^

^1^ Department of Psychosomatic Medicine and Psychotherapy, University Medical Center of the Johannes Gutenberg-University Mainz, Mainz, Germany

^2^ Preventive Cardiology and Preventive Medicine – Department of Cardiology, University Medical Center of the Johannes Gutenberg-University Mainz, Mainz, Germany

^3^ Center for Thrombosis and Hemostasis (CTH), University Medical Center of the Johannes Gutenberg-University Mainz, Mainz, Germany

^4^ Department of Cardiology – Cardiology I, University Medical Center of the Johannes Gutenberg-University Mainz, Mainz, Germany

^5^ Institute of Medical Biostatistics, Epidemiology and Informatics (IMBEI), University Medical Center of the Johannes Gutenberg-University Mainz

^6^ Institute of Clinical Chemistry and Laboratory Medicine, University Medical Center of the Johannes Gutenberg-University Mainz, Mainz, Germany

^7^ German Center for Cardiovascular Research (DZHK), partner site Rhine-Main, Mainz, Germany

^8^ Department of Ophthalmology, University Medical Center of the Johannes Gutenberg-University Mainz, Mainz, Germany

*Correspondence:*
M.Sc. Daniëlle Otten
Department of Psychosomatic Medicine and Psychotherapy
University Medical Center of the Johannes Gutenberg-University Mainz
Langenbeckstraße 1, 55131 Mainz, Germany
Phone: +49 (0)6131 17-7643
E-Mail: [Danielle.Otten@unimedizin-mainz.de](mailto:Danielle.Otten@unimedizin-mainz.de)

Supplementary Table 2a. Results of multiple logistic regression models of new onset of CVD, chronic obstructive lung disease, diabetes mellitus, cancer, and migraine on depressive symptoms at baseline for women.

|  | CVD | | | | Chronic obstructive  lung disease | | | Diabetes mellitus | | | Cancer | | | Migraine | | |
| --- | --- | --- | --- | --- | --- | --- | --- | --- | --- | --- | --- | --- | --- | --- | --- | --- |
|  |  | OR | CI | *p* | OR | CI | *p* | OR | CI | *p* | OR | CI | *p* | OR | CI | *p* |
|  |  |  |  |  |  |  |  |  |  |  |  |  |  |  |  |  |
| Depressive symptoms |  | 1.03 | 0.98-1.09 | .240 | **1.06** | 1.01-1.12 | .017 | 1.05 | 0.99-1.10 | .077 | 1.01 | 0.97-1.06 | .610 | **1.05** | 1.01-1.09 | .019 |
| *Sociodemographic* |  |  |  |  |  |  |  |  |  |  |  |  |  |  |  |  |
| Age |  | **1.07** | 1.04-1.09 | <.001 | 1.01 | 0.98-1.03 | .570 | 1.00 | 0.98-1.03 | .880 | **1.02** | 1.00-1.04 | .016 | **0.95** | 0.93-0.96 | <.001 |
| SES |  | **0.94** | 0.89-1.00 | .037 | 0.99 | 0.94-1.05 | .770 | 0.98 | 0.93-1.04 | .550 | 0.99 | 0.96-1.03 | .770 | **0.94** | 0.91-0.98 | .006 |
| Living with partner |  | 1.07 | 0.42-2.76 | .880 | 0.87 | 0.39-1.94 | .740 | 1.10 | 0.46-2.58 | .830 | 1.36 | 0.62-2.97 | .440 | 1.15 | 0.64-2.06 | .640 |
| Living alone |  | 0.86 | 0.31-2.39 | .770 | 1.05 | 0.43-2.55 | .910 | 1.03 | 0.41-2.62 | .950 | 1.25 | 0.54-2.92 | .600 | 0.80 | 0.40-1.61 | .540 |
| *Psychological* |  |  |  |  |  |  |  |  |  |  |  |  |  |  |  |  |
| Loneliness |  | 0.90 | 0.47-1.73 | .750 | 0.60 | 0.30-1.20 | .150 | 1.54 | 0.88-2.72 | .130 | 1.06 | 0.65-1.74 | .810 | 0.95 | 0.59-1.51 | .820 |
| *Metabolic* |  |  |  |  |  |  |  |  |  |  |  |  |  |  |  |  |
| BMI |  | **1.07** | 1.02-1.13 | .012 | **1.06** | 1.00-1.11 | .037 | **1.09** | 1.04-1.14 | <.001 | 1.01 | 0.97-1.06 | .610 | 1.03 | 0.98-1.07 | .240 |
| Dyslipidemia |  | 1.35 | 0.89-2.05 | .160 | 1.08 | 0.69-1.71 | .730 | 1.43 | 0.96-2.13 | .080 | 1.23 | 0.87-1.74 | .240 | 1.00 | 0.66-1.50 | .990 |
| Obesity |  | 0.74 | 0.38-1.45 | .380 | 1.09 | 0.56-2.11 | .800 | 1.15 | 0.63-2.12 | .650 | 0.99 | 0.56-1.72 | .960 | 0.67 | 0.36-1.23 | .200 |
| Blood glucose |  | 0.91 | 0.63-1.30 | .600 | 0.93 | 0.65-1.34 | .700 | **43.18** | 22.31-83.54 | <.001 | 1.07 | 0.83-1.39 | .600 | 0.88 | 0.64-1.20 | .410 |
| Hypertension |  | **1.80** | 1.14-2.83 | .011 | 1.07 | 0.69-1.67 | .760 | **1.78** | 1.13-2.80 | .013 | 1.08 | 0.77-1.53 | .640 | **0.64** | 0.44-0.94 | .023 |
| *Lifestyle* |  |  |  |  |  |  |  |  |  |  |  |  |  |  |  |  |
| Physical activity |  | 1.04 | 0.98-1.09 | .200 | 0.99 | 0.93-1.05 | .720 | 1.01 | 0.96-1.07 | .660 | 0.97 | 0.93-1.02 | .260 | 0.97 | 0.93-1.01 | .180 |
| Smoking |  | **2.54** | 1.60-4.03 | <.001 | **1.92** | 1.22-3.00 | .005 | 1.41 | 0.87-2.26 | .160 | 0.89 | 0.58-1.36 | .590 | 0.85 | 0.59-1.24 | .410 |
|  |  |  |  |  |  |  |  |  |  |  |  |  |  |  |  |  |
|  |  | Nagelkerke *R^2^*=.430 | | | Nagelkerke *R^2^*=.380 | | | Nagelkerke *R^2^*=.540 | | | Nagelkerke *R^2^*=.320 | | | Nagelkerke *R^2^*=.290 | | |

Note: OR=odds ratio; CI=confidence interval (2,5%-97,5%). For statistically significant predictors, the OR is printed in bold.
