# Supplementary material for: Depressive symptoms predict the incidence of common chronic diseases in women and men in a representative community sample
Source: Psychol Med. 2022 Apr 21;53(9):4172–80. doi: 10.1017/S0033291722000861 (PMC10317822; doi:10.1017/S0033291722000861)
Supplement: Supplementary file 1 [file S0033291722000861sup.zip › S0033291722000861sup003.docx]

**Depressive symptoms predict the incidence of common chronic diseases in women and men in a representative community sample**

Daniëlle Otten^1^, Mareike Ernst^1^, Antonia M. Werner^1^, Ana N. Tibubos^1^, Iris Reiner^1^, Elmar Brähler^1^, Jörg Wiltink^1^, Matthias Michal^1^, Markus Nagler^2^, Philipp S. Wild^2,3,7^, Thomas Münzel^4,7^, Jochem König^5^, Karl J. Lackner^6,7^, Norbert Peiffer^8^, Manfred E. Beutel^1^

^1^ Department of Psychosomatic Medicine and Psychotherapy, University Medical Center of the Johannes Gutenberg-University Mainz, Mainz, Germany

^2^ Preventive Cardiology and Preventive Medicine – Department of Cardiology, University Medical Center of the Johannes Gutenberg-University Mainz, Mainz, Germany

^3^ Center for Thrombosis and Hemostasis (CTH), University Medical Center of the Johannes Gutenberg-University Mainz, Mainz, Germany

^4^ Department of Cardiology – Cardiology I, University Medical Center of the Johannes Gutenberg-University Mainz, Mainz, Germany

^5^ Institute of Medical Biostatistics, Epidemiology and Informatics (IMBEI), University Medical Center of the Johannes Gutenberg-University Mainz

^6^ Institute of Clinical Chemistry and Laboratory Medicine, University Medical Center of the Johannes Gutenberg-University Mainz, Mainz, Germany

^7^ German Center for Cardiovascular Research (DZHK), partner site Rhine-Main, Mainz, Germany

^8^ Department of Ophthalmology, University Medical Center of the Johannes Gutenberg-University Mainz, Mainz, Germany

*Correspondence:*
M.Sc. Daniëlle Otten
Department of Psychosomatic Medicine and Psychotherapy
University Medical Center of the Johannes Gutenberg-University Mainz
Langenbeckstraße 1, 55131 Mainz, Germany
Phone: +49 (0)6131 17-7643
E-Mail: [Danielle.Otten@unimedizin-mainz.de](mailto:Danielle.Otten@unimedizin-mainz.de)

Supplementary Table 2b. Multiple logistic regression models of new onset of CVD, chronic obstructive lung disease, diabetes mellitus, cancer, and migraine on depressive symptoms at baseline for men.

|  | CVD | | | | Chronic obstructive  lung disease | | | Diabetes mellitus | | | Cancer | |  | Migraine | | |
| --- | --- | --- | --- | --- | --- | --- | --- | --- | --- | --- | --- | --- | --- | --- | --- | --- |
|  |  | OR | CI | p | OR | CI | p | OR | CI | p | OR | CI | p | OR | CI | p |
|  |  |  |  |  |  |  |  |  |  |  |  |  |  |  |  |  |
| Depressive symptoms |  | 1.04 | 1.00-1.09 | .065 | **1.07** | 1.02-1.12 | .008 | 1.03 | 0.99-1.08 | .170 | 1.03 | 0.99-1.08 | .110 | **1.07** | 1.02-1.13 | .010 |
| *Sociodemographic* |  |  |  |  |  |  |  |  |  |  |  |  |  |  |  |  |
| Age |  | **1.08** | 1.06-1.10 | <.001 | 1.02 | 1.00-1.04 | .070 | **0.98** | 0.96-1.00 | .021 | **1.08** | 1.06-1.10 | <.001 | **0.96** | 0.94-0.98 | .001 |
| SES |  | 1.00 | 0.97-1.04 | .850 | 0.98 | 0.94-1.02 | .360 | 0.97 | 0.93-1.00 | .051 | 1.02 | 0.99-1.06 | .130 | 0.98 | 0.93-1.03 | .400 |
| Living with partner |  | 0.73 | 0.33-1.62 | .440 | 1.57 | 0.38-6.51 | .540 | 0.55 | 0.24-1.25 | .150 | 2.40 | 0.58-9.84 | .230 | 0.74 | 0.26-2.09 | .560 |
| Living alone |  | 0.68 | 0.28-1.65 | .390 | 1.64 | 0.37-7.26 | .520 | 0.57 | 0.23-1.42 | .230 | 3.35 | 0.78-14.32 | .100 | 0.89 | 0.29-2.75 | .840 |
| *Psychological* |  |  |  |  |  |  |  |  |  |  |  |  |  |  |  |  |
| Loneliness |  | 1.25 | 0.73-2.15 | .410 | 1.59 | 0.87-2.92 | .130 | 0.95 | 0.51-1.77 | .880 | 0.78 | 0.44-1.40 | .400 | 1.48 | 0.77-2.86 | .240 |
| *Metabolic* |  |  |  |  |  |  |  |  |  |  |  |  |  |  |  |  |
| BMI |  | **1.09** | 1.04-1.14 | <.001 | 1.02 | 0.96-1.10 | .490 | **1.06** | 1.01-1.11 | .024 | 1.02 | 0.97-1.07 | .500 | 1.07 | 0.99-1.15 | .095 |
| Dyslipidemia |  | 1.21 | 0.92-1.61 | .180 | 1.46 | 1.00-2.14 | .053 | **1.41** | 1.03-1.92 | .032 | 1.11 | 0.85-1.45 | .450 | 0.88 | 0.56-1.39 | .580 |
| Obesity |  | 0.80 | 0.50-1.27 | .340 | 0.96 | 0.51-1.82 | .900 | 1.39 | 0.86-2.25 | .170 | 0.97 | 0.62-1.53 | .910 | 0.58 | 0.27-1.26 | .170 |
| Blood glucose |  | 1.13 | 0.93-1.37 | .220 | 0.87 | 0.64-1.19 | .390 | **57.28** | 33.83-96.99 | <.001 | 0.96 | 0.79-1.17 | .700 | **0.52** | 0.34-0.80 | .003 |
| Hypertension |  | 0.98 | 0.72-1.34 | .890 | 1.17 | 0.77-1.77 | .470 | **1.75** | 1.22-2.51 | .002 | 1.20 | 0.89-1.62 | .220 | 0.84 | 0.52-1.34 | .460 |
| *Lifestyle* |  |  |  |  |  |  |  |  |  |  |  |  |  |  |  |  |
| Physical activity |  | 1.01 | 0.98-1.05 | .530 | 1.02 | 0.98-1.07 | .350 | **0.96** | 0.92-1.00 | .031 | 1.00 | 0.96-1.04 | .980 | 1.00 | 0.95-1.06 | .890 |
| Smoking |  | **1.79** | 1.26-2.53 | .001 | 1.52 | 0.97-2.37 | .067 | 1.05 | 0.72-1.55 | .790 | 0.86 | 0.58-1.28 | .460 | 1.17 | 0.72-1.92 | .520 |
|  |  |  |  |  |  |  |  |  |  |  |  |  |  |  |  |  |
|  |  | Nagelkerke *R^2^*=.320 | | | Nagelkerke *R^2^*=.220 | | | Nagelkerke *R^2^*=.470 | | | Nagelkerke *R^2^*=.310 | | | Nagelkerke *R^2^*=.230 | | |

Note: OR=odds ratio; CI=confidence interval (2,5%-97,5%). For statistically significant predictors, the OR is printed in bold.
